# Hospital discharge communications during care transitions for patients with acute kidney injury: a cross-sectional study

**DOI:** 10.1186/s12913-016-1697-7

**Published:** 2016-08-30

**Authors:** Raquel C. Greer, Yang Liu, Deidra C. Crews, Bernard G. Jaar, Hamid Rabb, L. Ebony Boulware

**Affiliations:** 1 Division of General Internal Medicine, Johns Hopkins University School of Medicine, 2024 E. Monument Street, Room 2-626, Baltimore, MD 21287 USA; 2 Welch Center for Prevention, Epidemiology, and Clinical Research, Johns Hopkins Medical Institutions, Baltimore, USA; 3 Division of Nephrology, Johns Hopkins University School of Medicine, Baltimore, USA; 4 Department of Epidemiology, Johns Hopkins Bloomberg School of Public Health, Baltimore, USA; 5 Nephrology Center of Maryland, Baltimore, USA; 6 Division of General Internal Medicine, Duke University School of Medicine, Durham, NC USA

**Keywords:** Acute kidney injury, Transitions of care, Hospitalizations

## Abstract

**Background:**

High quality hospital discharge communications about acute kidney injury (AKI) could facilitate continuity of care after hospital transitions and reduce patients’ post-hospitalization health risks.

**Methods:**

We characterized the presence and quality (10 elements) of written hospital discharge communications (physician discharge summaries and patient instructions) for patients hospitalized with AKI at a single institution in 2012 through medical record review.

**Results:**

In 75 randomly selected hospitalized patients with AKI, fewer than half of physician discharge summaries and patient instructions documented the presence (*n* = 33, 44 % and *n* = 10, 13 %, respectively), cause (*n* = 32, 43 % and *n* = 1, 1 %, respectively), or course of AKI (*n* = 23, 31 %, discharge summary only) during hospitalization. Few provided recommendations for treatment and/or observation specific to AKI (*n* = 11, 15 and 6, 8 % respectively). In multivariable analyses, discharge communications containing information about AKI were most prevalent among patients with AKI Stage 3, followed by patients with Stage 2 and Stage 1 (adjusted percentages (AP) [95 % CI]: 84 % [39–98 %], 43 % [11–82 %], and 24 % [reference], respectively; p trend = 0.008). AKI discharge communications were also more prevalent among patients with known chronic kidney disease (CKD) versus those without (AP [95 % CI]: 92 % [51–99 %] versus 39 % [reference], respectively, *p* = 0.02) and among patients discharged from medical versus surgical services (AP [95 % CI]: 73 % [33–93 %] versus 23 % [reference], respectively, *p* = 0.01). Communications featured 4 median quality elements. Quality elements were greater in communications for patients with more severe AKI (Stage 3 (number of additional quality elements (β) [95 % CI]: 2.29 [0.87–3.72]), Stage 2 (β [95 % CI]: 0.62 [−0.65–1.90]) and Stage 1 (reference); p for trend = 0.002).

**Conclusions:**

Few hospital discharge communications in AKI patients described AKI or provided recommendations for AKI care. Improvements in the quality of hospital discharge communications to improve care transitions of patients with AKI are needed.

**Electronic supplementary material:**

The online version of this article (doi:10.1186/s12913-016-1697-7) contains supplementary material, which is available to authorized users.

## Background

Acute kidney injury (AKI) is increasingly common among hospitalized patients [1–4] and is associated with an increased risk of patients’ subsequent chronic kidney disease (CKD) incidence or progression to end stage renal disease as well as increased risks of in-hospital and long-term mortality [5–8]. Hospitalizations in which AKI occur often require medication adjustments and specialty care which can influence patients’ transition from inpatient to ambulatory care settings as well as their long-term care [9, 10]. Due to the risks associated with AKI, Kidney Disease Improving Global Outcomes (KDIGO) has issued clinical practice guidelines which recommend close follow-up of patients after an AKI event to monitor for early evidence of CKD or progression (i.e., monitoring for proteinuria or reduced estimated glomerular filtration rate) [11].

Inpatient health care providers’ acknowledgement and communication to patients’ ambulatory care providers about the occurrence of AKI during hospitalizations represents a crucial first step to ensuring patients receive recommended follow-up and monitoring after hospital discharge [11]. By communicating with ambulatory care providers (directly or via discharge summaries) about the occurrence, cause, course, and recommended follow-up care of AKI, inpatient providers can help ensure patients receive adequate follow up and management of their kidney function. Patient discharge instructions also help to ensure that patients receive follow up and are enabled to engage in disease self-management (i.e., avoidance of nephrotoxins and adherence to treatment for concomitant CKD risks).

Evidence suggests that awareness of AKI among health care providers and patients is generally suboptimal, and patients’ receipt of recommended follow-up care (including monitoring of kidney function) after a hospitalization with AKI is lacking [2, 12, 13]. Delayed or lack of adequate follow-up care after a hospitalization with AKI, particularly among patients with severe AKI, has been shown to contribute to patients’ poorer health outcomes [14]. A prior study assessed presence of documentation of kidney dysfunction in hospital discharge summaries to facilitate continuity of care of this high risk group [15]; however, the presence of hospital discharge communication about AKI to facilitate adequate follow-up has not been previously explored. We studied the presence and quality of hospital discharge communication about AKI from inpatient health care providers to patients’ ambulatory health care providers and to patients among individuals hospitalized for AKI in a teaching hospital. We also studied characteristics of hospitalizations associated with higher quality discharge communications.

## Methods

### Study overview

We conducted a cross-sectional manual review of inpatient hospital medical records to assess the presence and quality of hospital discharge communication about AKI.

### Study population

We examined serum creatinine measures among all adult patients (≥18 years-old) hospitalized in January, April, October and December of 2012 at Johns Hopkins Hospital in Baltimore, Maryland to assess whether patients experienced an episode of AKI during their hospitalizations. Following KDIGO criteria, we defined the presence of AKI as any increase in serum creatinine of ≥0.3 mg/dl within 48 h or increase in serum creatinine to ≥ 1.5 times baseline within the prior 7 days [11]. We further classified AKI severity as Stage 1 (an increase in serum creatinine of 1.5–1.9 times baseline or a ≥ 0.3 mg/dl increase), Stage 2 (an increase in serum creatinine of 2.0–2.9 times baseline), or Stage 3 (an increase in serum creatinine of ≥3.0 times baseline). Because we did not have access to patients’ pre-hospitalization serum creatinine, we considered patients’ lowest serum creatinine during their hospital stay to represent their baseline serum creatinine value. After we identified and characterized AKI among all patients, we randomly selected a subset of 25 patients from each of the three AKI stage groups (total 75 patients) for medical record review.

We excluded patients without a serum creatinine during hospitalization, patients requiring renal replacement therapy prior to their hospitalization; or patients who died during their admission.

### Data collection and measures

Each randomly selected patient’s electronic medical record underwent independent reviews by two trained research nurse abstractors. The nurses used a standardized form to abstract patients’: 1) demographics; 2) clinical characteristics (e.g., presence of CKD) documented in the discharge summary; 3) serum creatinine values during the hospitalization; 4) hospital course (e.g., length of stay or nephrology consultation during the hospitalization) 5) the presence of patients’ readmission within 30 days of hospital discharge; and 6) the presence and details (see below) of documentation of patients’ AKI in discharge summaries as well as documentation in patients’ discharge instructions intended to be printed out and given to patients at hospital discharge (Additional file 1). In the health system under study, the discharge summary and the patients’ discharge instructions are prepared by the admitting health care team. Nurses collected data using REDCap (Research Electronic Data Capture), a secure, web-based application designed to support data capture for research studies [16]. For each patient record, we examined all data entered independently by nurse abstractors for discrepancies in a manual review. When we identified differences, the two nurses re-consulted medical records and adjudicated. An investigator (R.G. or Y.L.) adjudicated any unresolved differences. We also assessed whether patients’ AKI was hospital or community acquired and we assessed the resolution of patients’ AKI episode. We considered AKI to be resolved prior to discharge if the final serum creatinine was <0.3 mg/dl above the nadir creatinine during the hospitalization.

### Presence of communication about AKI at hospital discharge

We defined communication about AKI at hospital discharge as being present if there was any notation or description of the AKI event in either (1) the discharge summaries written by inpatient providers describing the course of patients’ hospitalizations or (2) patients’ written discharge instructions. We used pre-specified terms (including ‘AKI’, ‘acute renal insufficiency’, or ‘creatinine increase’; see Additional file 1 for a complete list of terms) to identify communications pertaining to AKI.

### Assessment of quality of AKI communications at hospital discharge

We examined two primary aspects of AKI hospital discharge summaries: (1) whether summaries included complete descriptions of patients’ AKI course during the hospitalization, and (2) whether summaries included appropriate care plans (Table 1). To evaluate completeness of descriptions, we considered whether discharge summaries included information likely to be necessary for ambulatory care providers to provide adequate patient follow-up care for AKI including: (1) the occurrence of AKI; (2) the cause of AKI, and (3) the course of AKI during the hospitalization. To evaluate inclusion of appropriate care plans, we considered whether providers included (1) recommendations for follow-up appointments, (2) care plans for treatment and/or observation specific to the occurrence of AKI, and (3) identification of an ambulatory health provider to receive the discharge summary via fax, electronic communication, or mail. We also evaluated the type of ambulatory care provider (e.g., primary care, nephrology, or other specialist) for whom patients were scheduled or recommended to see in follow up care.

**Table 1:** Elements of AKI discharge communications assessed (*n* = 10)

Element	Type of communication	
	Discharge summary	Patient discharge instructions
Description of AKI event		
1) Documentation of occurrence of AKI	X	X
2) Identification of the cause of AKI (e.g., medication related, sepsis, etc.)	X	X
3) Description of the course of AKI during the hospitalization (e.g., serum creatinine prior to the start of AKI episode, peak serum creatinine, and discharge serum creatinine)	X	
Follow-up care plan		
4) Recommendations and timing of follow-up appointments or scheduled follow-up appointments	X	X
5) Recommendations for treatment and/or observation specific to the occurrence of AKI	X	X
6) Identification of an ambulatory health care provider (e.g., primary care physicians) to receive the discharge summary via fax, electronic communication, or mail.	X	

We examined whether patient discharge instructions provided patients with the necessary information to facilitate self-management and follow-up of AKI, including (1) the occurrence of AKI; (2) the cause of AKI, (3) care plans for treatment and/or observation, and (4) recommendations for follow-up with ambulatory care providers.

### Statistical analysis

We described the presence and quality of AKI hospital discharge communication in the patients’ written discharge materials and performed bivariate analyses (e.g., chi-square or t-tests) to assess differences in the presence and quality of AKI discharge communications according to patient (e.g., demographics, CKD status, comorbid medical conditions, AKI severity, AKI resolution, community vs. hospital acquired AKI, length of stay, intensive care unit stay during hospitalization, receipt of nephrology consultation) and system-level (e.g., discharging medical service) characteristics. We constructed a multivariable logistic regression model to assess patient and system level factors independently associated with the occurrence of AKI discharge communication in the discharge summary, patient discharge instructions, or both. We constructed a second multivariable linear regression model to assess whether the quality of patients’ discharge communications (i.e., the number of quality elements out of 10 total quality elements that are present in both the patients’ discharge summary (*n* = 6) and patient discharge instructions (*n* = 4)) varied by patient and system factors. Nephrology consultation data was missing for 13 % of patients. We performed subgroup analyses among patients with nephrology consultation data using the two multivariable models described above to evaluate the independent association of nephrology consultation with the occurrence and quality of discharge communication about AKI. We performed all statistical analyses using Stata, version 11 (StataCorp, www.stata.com).

## Results

### Participant selection

Among 11,372 patients who were hospitalized during the 4-month time period, we excluded those without a serum creatinine (*n* = 392), patients requiring chronic renal replacement therapies prior to their hospitalization (*n* = 512); and patients who died during hospitalization (*n* = 179). Of the remaining 10,289 patients, we identified 2859 patients with AKI. We stratified patients according to AKI severity (Stage 1 (*n* = 2191), Stage 2 (*n* = 474), or Stage 3 (*n* = 194)), and selected a random sample of 25 patients from within each stratum for a manual review of their medical records.

### Participant characteristics

Among the 75 randomly selected patients with AKI, the mean age on admission was 56 years old. A majority were female (*n* = 46, 61 %) and nearly half were African American (*n* = 36, 48 %). Over half (*n* = 44, 59 %) of patients had ≥1 medical risk factor for AKI at the time of admission and the median Charlson comorbidity index score was 2 (Interquartile range: 1–4). Patients were hospitalized a median of 9 days and were most frequently discharged from Medicine (*n* = 34, 45 %) followed by Surgical (*n* = 26, 35 %) inpatient services. Patients were admitted most commonly for infections (*n* = 16, 21 %), followed by diseases or disorders related to the circulatory system (*n* = 15, 20 %), the respiratory system (*n* = 11, 15 %), malignancy (*n* = 10, 13 %), or the digestive system (*n* = 8, 11 %). The majority (*n* = 46, 61 %) of patients had hospital-acquired compared to community-acquired AKI. Twenty percent (*n* = 13) of patients with AKI had a nephrology consultation during their hospitalization.

Patient demographics and clinical characteristics were similar between AKI Stage groups; except for CKD, which was not present among patients with Stage 3 AKI. The median length of stay varied by AKI severity with longer stays among patients with Stage 3 AKI (15 days), followed by Stage 2 (10 days) and Stage 1 (6 days) AKI. The occurrence of nephrology consultations during the hospitalization also varied by AKI severity with greater occurrence among patients with Stage 3 AKI (*n* = 9, 43 %), followed by Stage 2 (*n* = 3, 15 %) and Stage 1 (*n* = 1, 4 %) AKI (Table 2).

**Table 2:** Patient characteristics (*n* = 75)

Patient characteristics	Total (*n* = 75)	Stage 1 (*N* = 25)	Stage 2 (*N* = 25)	Stage 3 (*N* = 25)	*P* value
Age, mean years (SD)	56 (19)	62 (18)	53 (21)	52 (18)	0.16
Female, n (%)	46 (61)	14 (56)	14 (56)	18 (72)	0.41
Race, n (%)					0.35
White	36 (48)	12 (48)	11 (44)	13 (52)	
Black	36 (48)	13 (52)	13 (52)	10 (40)	
Asian	1 (1)	0 (0)	1 (4)	0 (0)	
Other	2 (3)	0 (0)	0 (0)	2 (8)	
Diabetes, n (%)	16 (21)	5 (20)	6 (24)	5 (20)	0.92
Hypertension, n (%)	33 (44)	14 (56)	11 (44)	8 (32)	0.23
Chronic kidney disease, n (%)	11 (15)	5 (20)	6 (24)	0 (0)	0.04
Coronary artery disease, n (%)	16 (21)	5 (20)	5 (20)	6 (24)	0.92
Congestive heart failure, n (%)	11 (15)	2 (8)	2 (8)	7 (28)	0.07
Charlson comorbidity score, median (IQR)	2 (1–4)	2 (1–5)	2 (2–4)	2 (0–3)	0.25
Length of stay, median days (IQR)	9 (6–16)	6 (4–7)	10 (8–16)	15 (7–35)	<0.001
Admission diagnosis categories					
Infectious	16 (21)	3 (12)	5 (20)	8 (32)	0.22
Circulatory	15 (20)	7 (28)	5 (20)	3 (12)	0.37
Respiratory	11 (15)	5 (20)	3 (12)	2 (12)	0.65
Malignancy	10 (13)	4 (16)	4 (16)	2 (8)	0.63
Digestive	8 (11)	2 (8)	5 (20)	1 (4)	0.16
Renal	6 (8)	3 (12)	2 (8)	1 (4)	0.58
Endocrine/metabolic	2 (3)	0 (0)	0 (0)	2 (8)	0.13
Other	7 (9)	1 (4)	1 (4)	5 (20)	0.08
Discharge Service, n (%)					0.26
Medical	34 (45)	12 (48)	13 (52)	9 (36)	
Surgical	26 (35)	10 (40)	5 (20)	11 (44)	
Oncology	13 (17)	2 (8)	7 (28)	4 (16)	
Other	2 (3)	1 (4)	0 (0)	1 (4)	
ICU stay during hospitalization, n (%)	23 (31)	6 (24)	5 (20)	12 (48)	0.067
Nadir creatinine, mean (SD) (mg/dl)^a^	1.01 (0.90)	1.28 (1.25)	1.00 (0.81)	0.76 (0.32)	0.12
Discharge creatinine, mean (SD) (mg/dl)	1.39 (1.09)	1.45 (1.30)	1.30 (1.14)	1.42 (0.82)	0.87
Hospital-acquired AKI, n (%)	46 (61)	14 (56)	14 (56)	18 (72)	0.41
Nephrology Consult^b^, n (%)	13 (20)	1 (4)	3 (15)	9 (43)	0.004
30 day hospital readmission, n (%)	14 (19)	7 (28)	4 (16)	3 (12)	0.32

^a^Nadir serum creatinine – lowest serum creatinine during hospital admission; Abbreviations: ICU- intensive care unit

^b^Assessed among 65 patients with consult information available in the electronic medical record

### Presence and quality of communication about AKI at hospital discharge

#### Description of AKI event

**Fig. 1:**
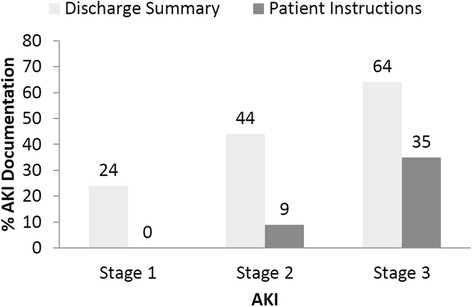
Presence of AKI Documentation in the Discharge Summary and Written Patient Instructions

Less than half (*n* = 33, 44 %) of discharge summaries targeted for ambulatory care providers mentioned the presence of AKI during hospitalization, and few (*n* = 10, 13 %) patient instructions mentioned the AKI event. Rates of AKI communication in the discharge summary or patient instructions were lowest for patients with Stage 1 AKI (*n* = 6, 24 % for discharge summaries and none for patient instructions) and highest for patients with Stage 3 AKI (*n* = 16, 64 % for discharge summaries and *n* = 8, 35 % for patient instructions) (Fig. 1).

Among the 33 discharge summaries in which inpatient provider communications about AKI were present, nearly all (*n* = 32, 97 %) mentioned the suspected cause of patients’ AKI event; however a third (*n* = 11, 33 %) lacked some description of the course of AKI (e.g., discharge serum creatinine). The suspected cause of AKI was infrequently mentioned in the patient instructions (*n* = 1, 1 %) (Table 3).

**Table 3:** Quality of AKI Communication at Hospital Discharge (10 quality elements)

	Type of communication N (%)	
Element	Discharge summary	Patient discharge instructions
Description of AKI event		
1) Documentation of occurrence of AKI	33 (44)	10 (13)
2) Identification of the cause of AKI	32 (43)	1 (1)
3) Description of the course of AKI during the hospitalization	23 (31)	
Follow-up care plan		
1) Recommendations for follow-up appointments or scheduled follow-up appointments	60 (80)	67 (89)
2) Recommendations for treatment and/or observation specific to the occurrence of AKI	11 (15)	6 (8)
3) Identification of an ambulatory health care provider to receive the discharge summary via fax, electronic communication, or mail.	58 (77)	

#### Follow-up care plans

The vast majority of patients had follow-up appointment recommendations documented in the discharge summary (*n* = 60, 80 %) or patient instructions (*n* = 67, 89 %). Nearly half (*n* = 33, 44 %) had recommendations for follow-up with their primary care provider, while fewer had recommendations to follow up with a cardiologist (*n* = 10, 13 %) or a nephrologist (*n* = 5, 7 %). Follow-up appointments were scheduled within 30 days from discharge for the majority (*n* = 63, 84 %) of the patients. Among the 75 patients, a majority (*n* = 58, 77 %) of patients had scheduled appointments within 2 weeks, and nearly half (*n* = 35, 47 %) had scheduled appointments within one week. Few patients had documented recommendations for treatment (e.g., avoidance of nephrotoxins, discontinuation of medications) and/or observation (e.g., follow-up lab tests) related to their AKI in either the discharge summary (*n* = 11, 15 %) or patient instructions (*n* = 6, 8 %). Follow-up care plans (type of provider and timing of appointment) were similar in those with resolved versus unresolved AKI; however, documented recommendations for follow-up laboratory tests related to AKI in the written discharge materials were greater among patients whose AKI was unresolved versus resolved (*n* = 8, 33 % versus *n* = 4, 8 %, *p* = 0.005) prior to discharge. Patients with documented CKD in the discharge summary were more likely to have scheduled follow-up with a nephrologist, compared to patients without CKD (*n* = 5, 45 % versus *n* = 0, 0 %, respectively, *p* < 0.001). There were no statistically significant differences in recommendations for nephrology follow-up by AKI severity or by resolution of AKI prior to discharge. Nearly a quarter (*n* = 17, 23 %) of patients’ discharge summaries did not have a referring provider identified to receive a copy of the discharge summary.

Among the 75 patients, none included all 10 quality elements we assessed in the discharge summary and the patient instructions, 5 (7 %) included 8–9 elements, 23 (31 %) included 5–7 elements, 29 (39 %) included 3–4 elements, and 18 (24 %) included 2 or fewer elements. The median (interquartile range) number of quality elements present in the discharge materials was 3 (3–6).

### Determinants of the occurrence and quality of AKI discharge communications

In multivariable analyses, the prevalence of communication about AKI in inpatient providers’ discharge summaries was greatest among patients with AKI Stage 3, followed by patients with Stage 2 and Stage 1 AKI (adjusted percentages (AP) [95 % CI]: 84 % [39–98 %], 43 % [11–82 %], and 24 % [reference], respectively; p trend = 0.008). The prevalence of communication about AKI in discharge summaries was also greater among patients discharged from medical services compared to surgical services (AP [95 % CI]: 73 % [33–93 %] versus 23 % [reference], *p* = 0.01) and among patients with CKD compared to those patients without CKD (AP [95 % CI]: 92 % [51–99 %] versus 39 % [reference], *p* = 0.02). The number of quality elements present in the discharge summary and discharge instructions was also greater among those with more severe AKI (AKI Stage 3 (β [95 % CI]: 2.29 [0.87–3.72]), AKI Stage 2 (β [95 % CI]: 0.62 [−0.65–1.90]) and AKI stage 1 (reference); test for trend across all three stages *p* = 0.002) (Table 4).

**Table 4:** Predictors of the Occurrence and Quality of AKI in Discharge Communication

Patient characteristics	Presence of AKI in discharge communication (*N* = 75)		Quality of AKI discharge communication (*N* = 75)	
	Adjusted %^a^ (95 % CI)	*P* value	β^ab^ (95 % CI) Quality elements	*P* value
Age		0.65		0.47
≥ 65 years	42 (14–76)		0.39 (−0.69, 1.47)	
< 65 years	50 (Ref)		Ref	
Sex				
Male	50 (22–78)	0.88	−0.38 (−1.34, 0.58)	0.44
Female	48 (Ref)		Ref	
Race				
African American	62 (31–86)	0.11	0.48 (−0.52, 1.48)	0.34
White/Asian/Other	36 (Ref)		Ref	
Diabetes				
Yes	29 (06–71)	0.52	−0.26 (−1.61, 1.08)	0.70
No	42 (Ref)		Ref	
Congestive heart failure				
Yes	59 (17–91)	0.41	0.93 (−0.57, 2.42)	0.22
No	39 (Ref)		Ref	
Chronic kidney disease				
Yes	92 (51–99)	0.02	1.04 (−0.48, 2.56)	0.18
No	39 (Ref)		Ref	
AKI stage				
3	84 (39–98)	0.009^*^	2.29 (0.87, 3.72)	0.002^*^
2	43 (11–82)	0.35	0.62 (−0.65, 1.90)	0.33
1	24 (Ref)		Ref	
Unresolved AKI on discharge				
Yes	47 (18–79)	0.51	0.40 (−0.67, 1.48)	0.46
No	35 (Ref)		Ref	
Discharge service				
Medical	73 (33–93)	0.01	0.87 (−0.21, 1.94)	0.11
Surgery	23 (Ref)		Ref	
Length of stay (days)				
High (13+)	49 (10–89)	0.74	−1.36 (−3.00, 0.28)	0.10
Middle (7–12)	50 (15–85)	0.65	−0.27 (−1.56, 1.01)	0.67
Low (1–6)	40 (Ref)		Ref	
Intensive care unit stay				
Yes	49 (13–85)	0.91	0.54 (−0.72, 1.79)	0.40
No	46 (Ref)		Ref	

*p for trend in adjusted analysis < 0.05

^a^Adjusted for age, race, sex, diabetes, congestive heart failure, chronic kidney disease, AKI stage, AKI resolution, discharge service, length of stay and intensive care unit stay

^b^β are for the number of quality elements present in both the discharge summary and the patient discharge instructions

*Abbreviations*: *AKI* acute kidney injury

There was no difference in the presence and quality of documentation between patients with community versus hospital acquired AKI or by patients’ recovery of kidney function prior to discharge. While there was a trend towards lower hospital readmission rates among those with AKI discharge communication compared to those without, the difference was not statistically significant (AP [95 % CI] 15 % [2–59 %] versus 24 % [reference], *p* = 0.14).

In separate multivariable analyses among the subgroup of patients with nephrology consultation data (*n* = 65), the prevalence of communication about AKI in discharge summaries and the number of quality elements present in the discharge summary was greater among patients who had a nephrology consultation compared to those patients who did not obtain a nephrology consultation during their inpatient admission (AP [95 % CI]: 99 % [71–100 %] versus 33 % [reference], *p* = 0.01; (β [95 % CI]: 1.87 [0.41–3.32], *p* = 0.01, respectively).

## Discussion

Our detailed characterization of the presence and quality of physicians’ discharge communications about AKI during patients’ transitions from inpatient to outpatient settings uncovered numerous communication deficiencies. Communications about AKI during hospital stays were infrequent in both physicians’ discharge summaries and in patients’ discharge instructions. Further, they were less prevalent and/or of lower quality among patients with less severe AKI, among patients who were discharged from surgical services, among patients without a history of CKD, and among patients without a nephrology consultation during their inpatient stay. Most discharge summaries and patient instructions lacked sufficient detail regarding the AKI event, and numerous patients had either no identified provider to receive communications or poor nephrology follow up.

To our knowledge, this is the first study to characterize deficiencies in hospital discharge communications to both providers and patients that could adversely affect patients’ post hospital care. Prior studies have described the suboptimal follow-up care (including inadequate monitoring of kidney function) of patients after a hospitalization with AKI [2, 13]. However, little attention has been paid to how hospital providers’ communications facilitate patients’ successful transitions to ambulatory care settings. Our findings provide important insights for efforts to identify strategies to improve care and outcomes among survivors of AKI [17].

Our findings of lower communication rates about AKI when AKI was less severe suggest hospital providers may not recognize less severe AKI. Dissemination of clinical practice guidelines to enhance hospital providers’ knowledge and awareness of AKI could heighten their awareness of AKI and prompt them to include AKI in discharge communications more frequently. Decision support tools (such as electronic medical alerts or checklists) have been shown to improve care transitions [18] and hospital providers’ recognition of AKI [19–22], and they could also enhance providers’ focus on AKI in discharge communications. These tools could be particularly important for non-medical specialists who may have substantially less experience managing AKI. Greater oversight by the consulting nephrology team regarding the provision of information about AKI and follow-up recommendations within the discharge materials may also be beneficial. In our study, the greater occurrence and higher quality discharge communication about AKI among patients who had a nephrology consultation during their hospitalization suggests that greater involvement of nephrology care during the admission and in the discharge planning may improve care transitions for patients with AKI.

The low rates of nephrology follow-up care at hospital discharge observed in our study and in prior studies [23, 24] may be due to hospital providers’ poor understanding of the long-term CKD risks associated with AKI events and/or uncertainty of which patients should be referred for subsequent nephrology care. Studies providing evidence on which patients would benefit most from early nephrology evaluation and management following a hospitalization with AKI are needed. While we did not observe a statistically significant association between the presence of AKI communication and hospital readmission in this small study, we did observe a trend towards lower hospital readmission rates among patients with AKI discharge communication compared to those without AKI discharge communication, suggesting further studies to understand the potential impact of discharge communications on this important clinical outcome are warranted.

The rates of identification of an ambulatory provider to receive the discharge summary observed in our study was suboptimal, and may in part be due to fragmented care structures in which ambulatory providers are not always affiliated directly with the admitting hospital. Greater integration of health care systems could facilitate timely discharge communication about AKI to ambulatory providers and timely follow-up of AKI after hospital discharge. Additionally, implementation of multidisciplinary discharge planning teams could also aid communication about AKI during care transitions, particularly if teams are trained to communicate (both verbally and in writing) with ambulatory primary care providers and nephrologists about AKI, to provide patient education about AKI, to facilitate medication reconciliation, and to schedule ambulatory follow-up appointments.

The limitations of our study deserve mention. First, we assessed documentation among a small number of randomly selected patients receiving care in a single teaching hospital over one year period, which may potentially limit the generalizability of our results. AKI communication rates may differ at other hospitals or in other geographic regions. However, our findings are consistent with other studies demonstrating deficiencies with AKI recognition and follow-up care [2, 13, 25–27]. New clinical practice guidelines for AKI emerged in 2012 and the cross-sectional design of our study limits our ability to assess changes in rates of discharge communication about AKI over time [11]. Longitudinal studies elucidating time related trends in rates of AKI discharge communication in relation to release of the new guidelines could help clarify whether patterns of care are improving as greater awareness about AKI risks evolve among the general medical community. Second, we assessed only written discharge materials. It is possible other types of communication (e.g., telephone or electronic communication with ambulatory providers or in-person discussions with patients) occurred at the time of patients’ hospital discharge. Third, because not all patients received their outpatient care in the health system under study, we established the nadir creatinine during the hospital stay as a reflection of patients’ baseline serum creatinines. It is possible this approach could have resulted in bidirectional misclassification of patients’ AKI diagnosis and severity [28, 29]. Nonetheless, we believe it is common in the US, where many health systems are not integrated, for clinicians to make judgements about the presence of AKI with limited outpatient data available. Thus, we believe our assessments of AKI and measurement of clinical care in the presence of AKI are likely to reflect those made in similar real-world settings. Finally, we were unable to ascertain whether primary care providers followed up on reported AKI. Future studies are needed to assess whether improved discharge communication about AKI improves the provision of appropriate ambulatory follow-up and reduces patients’ post-hospitalization health risks.

## Conclusions

In summary, communication of AKI events between hospital providers to patients and their ambulatory providers during hospital discharge transitions was low and the overall quality of the documentation was poor. Efforts to enhance awareness of AKI and to prompt improved communication at the time of hospital discharge, may improve care transitions for these high risk patients.

## Additional file

Additional file 1: Medical Record Review Components. (PDF 77 kb)
