# Supplementary material for: Hospital discharge communications during care transitions for patients with acute kidney injury: a cross-sectional study
Source: BMC Health Serv Res. 2016 Aug 30;16(1):449. doi: 10.1186/s12913-016-1697-7 (PMC5006255; doi:10.1186/s12913-016-1697-7)
Supplement: Additional file 1: — Medical Record Review Components. (PDF 77 kb) [file 12913_2016_1697_MOESM1_ESM.pdf]

Additional file 1: Medical Record Review Components

**Discharge Summary**
**Patient Demographics**<br>Patient age on admission<br>Gender<br>Race/ethnicity
**Clinical Characteristics**<br>AKI risk factors (e.g., chronic kidney disease, diabetes, hypertension, coronary artery disease, congestive heart failure)<br>Other medical conditions (e.g., stroke, malignancy, liver disease)
**Hospital Course**<br>Length of stay (days)<br>Admitting and discharge service<br>Transfers during hospitalization (count)<br>Intensive care unit stay during hospitalization<br>Primary admitting diagnosis<br>Discharge medications<br>Dialysis requirement (i.e., hospitalization/discharge)<br>Patient Disposition (e.g., home, skilled nursing facility, etc)<br>Readmission within 30 days
**AKI Documentation**<br>Terminology Used to Document AKI (i.e., kidney/renal injury, kidney/renal insufficiency, kidney/renal failure, creatinine increase/rise/bump, eGFR decline/decrease, prerenal, decreased/low urine output, or other)<br>Cause of AKI (e.g., Contrast media administration, medication-related, sepsis, volume contraction, overdiuresis, cardiogenic shock, etc.)<br>Course of AKI (i.e., nadir/baseline, peak, and discharge creatinine)
**Follow-up Care**<br>Appointment(s) (i.e., timing and provider type)<br>AKI related laboratory tests (i.e., BMP, creatinine, or potassium)<br>Physician listed to receive discharge summary
**Patient Discharge Worksheet**
**AKI Documentation**<br>Terminology Used to Document AKI<br>Cause of AKI
**Follow-up Care**<br>Follow-up Appointment (i.e. timing and provider type)<br>Follow-up laboratory tests related to AKI<br>Patient education about AKI (e.g., avoid NSIADS, medication adjustment/discontinuation, hydration, other)
